# *N*-(3,5-Dichloro­phen­yl)-4-methyl­benzene­sulfonamide

**DOI:** 10.1107/S1600536809034801

**Published:** 2009-09-05

**Authors:** B. Thimme Gowda, Sabine Foro, P. G. Nirmala, Hartmut Fuess

**Affiliations:** a Department of Chemistry, Mangalore University, Mangalagangotri 574 199, Mangalore, India; b Institute of Materials Science, Darmstadt University of Technology, Petersenstrasse 23, D-64287 Darmstadt, Germany

## Abstract

In the crystal structure of the title compound, C_13_H_11_Cl_2_NO_2_S, the conformation of the N—C bond in the C—SO_2_—NH—C segment is *gauche* with respect to the SO bonds. The two benzene rings are tilted by 79.6 (1)° relative to each other. In the crystal, inversion dimers linked by pairs of N—H⋯O hydrogen bonds occur.

## Related literature

For the preparation of the title compound, see: Shetty & Gowda (2005 ▶). For background literature, see: For a study of the effect of substituents on the crystal structures of *N*-(ar­yl)-aryl­sulfonamides, see: Gowda *et al.* (2008 ▶, 2009**a* ▶,b* ▶). For bond parameters in related aryl sulfonamides, see: Gelbrich *et al.* (2007 ▶); Perlovich *et al.* (2006 ▶).
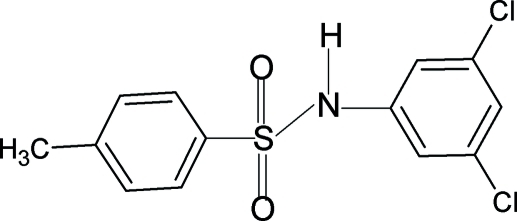


## Experimental

### 

#### Crystal data

C_13_H_11_Cl_2_NO_2_S
*M* *_r_* = 316.19
Monoclinic, 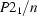

*a* = 6.7388 (8) Å
*b* = 8.9627 (8) Å
*c* = 22.944 (2) Å
β = 91.801 (8)°
*V* = 1385.1 (2) Å^3^
*Z* = 4
Cu *K*α radiation
μ = 5.61 mm^−1^
*T* = 299 K
0.42 × 0.35 × 0.13 mm

#### Data collection

Enraf–Nonius CAD-4 diffractometer
Absorption correction: ψ scan (North *et al.*, 1968 ▶) *T*_min_ = 0.140, *T*_max_ = 0.482
3363 measured reflections
2424 independent reflections
2049 reflections with *I* > 2σ(*I*)
*R*_int_ = 0.104
3 standard reflections frequency: 120 min intensity decay: 1.0%

#### Refinement

*R*[*F* ^2^ > 2σ(*F* ^2^)] = 0.056
*wR*(*F* ^2^) = 0.236
*S* = 1.11
2424 reflections
176 parameters
H atoms treated by a mixture of independent and constrained refinement
Δρ_max_ = 0.48 e Å^−3^
Δρ_min_ = −0.64 e Å^−3^

### 

Data collection: *CAD-4-PC* (Enraf–Nonius, 1996 ▶); cell refinement: *CAD-4-PC*; data reduction: *REDU4* (Stoe & Cie, 1987 ▶); program(s) used to solve structure: *SHELXS97* (Sheldrick, 2008 ▶); program(s) used to refine structure: *SHELXL97* (Sheldrick, 2008 ▶); molecular graphics: *PLATON* (Spek, 2009 ▶); software used to prepare material for publication: *SHELXL97*.

## Supplementary Material

Crystal structure: contains datablocks I, global. DOI: 10.1107/S1600536809034801/bv2124sup1.cif

Structure factors: contains datablocks I. DOI: 10.1107/S1600536809034801/bv2124Isup2.hkl

Additional supplementary materials:  crystallographic information; 3D view; checkCIF report

## Figures and Tables

**Table 1:** Hydrogen-bond geometry (Å, °)

*D*—H⋯*A*	*D*—H	H⋯*A*	*D*⋯*A*	*D*—H⋯*A*
N1—H1*N*⋯O1^i^	0.87 (5)	2.02 (6)	2.888 (5)	176 (5)

Symmetry code: (i) 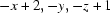.

**Fig. 1.**
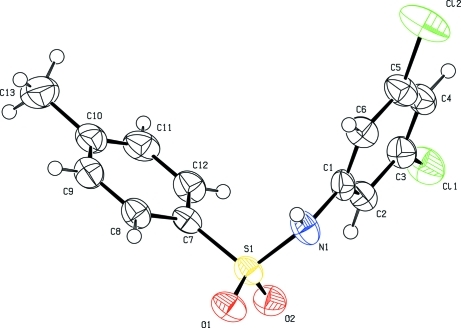
Molecular structure of (I), showing the atom labelling scheme and displacement ellipsoids are drawn at the 50% probability level.

**Fig. 2.**
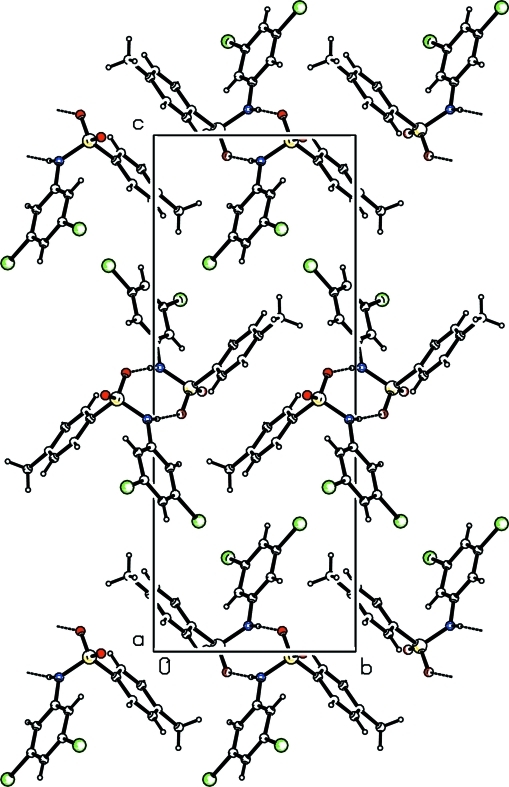
Molecular packing of (I) with hydrogen bonding shown as dashed lines.

## supplementary crystallographic information

### Comment

As part of a study of the substituent effects on the crystal structures of *N*-(aryl)-arylsulfonamides (Gowda *et al.*, 2008; 2009*a, b*), in the present work, the crystal structure of 4-methyl-*N*-(3,5-dichlorophenyl)benzenesulfonamide (I) has been determined. The conformation of the N—C bond in the C—SO_2_—NH—C segment of the structure has "*trans*" and "*gauche*" torsions with respect to the SO bonds (Fig. 1). The molecule is bent at the S atom with the C—SO_2_—NH—C torsion angle of 69.3 (4)° compared to the values of -51.6 (3)° and 68.3 (2)°, respectively, for 4-methyl-*N*-(phenyl)benzenesulfonamide (II)(Gowda *et al.*, 2009*b*) and *N*-(3,5-dichlorophenyl)-benzenesulfonamide (III) (Gowda *et al.*, 2008). The two benzene rings in (I) are tilted relative to each other by 79.6 (1)°, compared to the values of 68.4 (1)° for the compound II and 57.0 (1)° for III.

The other bond parameters in (I) are similar to those observed in (II) (Gowda *et al.*, 2009*b*), (III) (Gowda *et al.*, 2008), 4-methyl-*N*-(3,4-dimethylphenyl)benzenesulfonamide (Gowda *et al.*, 2009*a*) and other aryl sulfonamides (Perlovich *et al.*, 2006; Gelbrich *et al.*, 2007). The packing of molecules *via* N—H···O(S) hydrogen bonds (Table 1) into supramolecular structure is shown in Fig. 2.

### Experimental

The solution of toluene (10 cc) in chloroform (40 cc) was treated dropwise with chlorosulfonic acid (25 cc) at 0 ° C. After the initial evolution of hydrogen chloride subsided, the reaction mixture was brought to room temperature and poured into crushed ice in a beaker. The chloroform layer was separated, washed with cold water and allowed to evaporate slowly. The residual 4-methylbenzenesulfonylchloride was treated with 3,5-dichloroaniline in the stoichiometric ratio and boiled for 15 minutes. The reaction mixture was then cooled to room temperature and added to ice cold water (100 cc). The resultant 4-methyl-*N*-(3,5-dichlorophenyl)benzenesulfonamide was filtered under suction and washed thoroughly with cold water. It was then recrystallized to constant melting point from dilute ethanol. The purity of the compound was checked and characterized by recording its infrared and NMR spectra (Shetty & Gowda, 2005). The prism like colourless single crystals used in X-ray diffraction studies were grown in ethanolic solution by slow evaporation at room temperature.

### Refinement

The H atom of the NH group was located in a difference map and its position refined [N—H = 0.87 (5) Å]. The other H atoms were positioned with idealized geometry using a riding model [C—H = 0.93–0.96 Å]. All H atoms were refined with isotropic displacement parameters (set to 1.2 times of the *U*_eq_ of the parent atom).

Two reflections (-5 1 9 and -3 2 11) were omitted from the refinement as a statistical analysis showed that they were anomalous.

### Crystal data

**Table:**

C_13_H_11_Cl_2_NO_2_S	*F*(000) = 648
*M**_r_* = 316.19	*D*_x_ = 1.516 Mg m^−^^3^
Monoclinic, *P*2_1_/*n*	Cu *K*α radiation, λ = 1.54180 Å
Hall symbol: -P 2yn	Cell parameters from 25 reflections
*a* = 6.7388 (8) Å	θ = 3.9–18.2°
*b* = 8.9627 (8) Å	µ = 5.61 mm^−^^1^
*c* = 22.944 (2) Å	*T* = 299 K
β = 91.801 (8)°	Prism, colourless
*V* = 1385.1 (2) Å^3^	0.42 × 0.35 × 0.13 mm
*Z* = 4	

### Data collection

**Table:**

Enraf–Nonius CAD-4 diffractometer	2049 reflections with *I* > 2σ(*I*)
Radiation source: fine-focus sealed tube	*R*_int_ = 0.104
graphite	θ_max_ = 66.9°, θ_min_ = 3.9°
ω/2θ scans	*h* = −7→2
Absorption correction: ψ scan (North *et al.*, 1968)	*k* = −10→0
*T*_min_ = 0.140, *T*_max_ = 0.482	*l* = −27→27
3363 measured reflections	3 standard reflections every 120 min
2424 independent reflections	intensity decay: 1.0%

### Refinement

**Table:**

Refinement on *F*^2^	Primary atom site location: structure-invariant direct methods
Least-squares matrix: full	Secondary atom site location: difference Fourier map
*R*[*F*^2^ > 2σ(*F*^2^)] = 0.056	Hydrogen site location: inferred from neighbouring sites
*wR*(*F*^2^) = 0.236	H atoms treated by a mixture of independent and constrained refinement
*S* = 1.11	*w* = 1/[σ^2^(*F*_o_^2^) + (0.1586*P*)^2^ + 1.125*P*] where *P* = (*F*_o_^2^ + 2*F*_c_^2^)/3
2424 reflections	(Δ/σ)_max_ = 0.011
176 parameters	Δρ_max_ = 0.48 e Å^−^^3^
0 restraints	Δρ_min_ = −0.64 e Å^−^^3^

### Special details

**Table:**

Geometry. All e.s.d.'s (except the e.s.d. in the dihedral angle between two l.s. planes) are estimated using the full covariance matrix. The cell e.s.d.'s are taken into account individually in the estimation of e.s.d.'s in distances, angles and torsion angles; correlations between e.s.d.'s in cell parameters are only used when they are defined by crystal symmetry. An approximate (isotropic) treatment of cell e.s.d.'s is used for estimating e.s.d.'s involving l.s. planes.
Refinement. Refinement of *F*^2^ against ALL reflections. The weighted *R*-factor *wR* and goodness of fit *S* are based on *F*^2^, conventional *R*-factors *R* are based on *F*, with *F* set to zero for negative *F*^2^. The threshold expression of *F*^2^ > σ(*F*^2^) is used only for calculating *R*-factors(gt) *etc*. and is not relevant to the choice of reflections for refinement. *R*-factors based on *F*^2^ are statistically about twice as large as those based on *F*, and *R*- factors based on ALL data will be even larger.

### Fractional atomic coordinates and isotropic or equivalent isotropic displacement parameters (Å^2^)

**Table:**

	*x*	*y*	*z*	*U*_iso_*/*U*_eq_	
C1	0.6639 (6)	0.0125 (4)	0.60350 (17)	0.0403 (9)	
C2	0.4841 (6)	0.0772 (5)	0.61451 (17)	0.0442 (9)	
H2	0.4215	0.1394	0.5872	0.053*	
C3	0.3986 (6)	0.0473 (5)	0.66742 (18)	0.0441 (9)	
C4	0.4832 (7)	−0.0443 (5)	0.70890 (18)	0.0470 (10)	
H4	0.4223	−0.0636	0.7439	0.056*	
C5	0.6627 (7)	−0.1064 (5)	0.69595 (19)	0.0496 (10)	
C6	0.7556 (7)	−0.0807 (5)	0.64425 (18)	0.0454 (9)	
H6	0.8771	−0.1249	0.6369	0.054*	
C7	0.9133 (6)	0.3137 (4)	0.54905 (17)	0.0393 (9)	
C8	1.1092 (6)	0.3369 (5)	0.53436 (18)	0.0430 (9)	
H8	1.1630	0.2845	0.5036	0.052*	
C9	1.2229 (7)	0.4380 (5)	0.56558 (19)	0.0495 (10)	
H9	1.3544	0.4532	0.5560	0.059*	
C10	1.1436 (8)	0.5185 (5)	0.6117 (2)	0.0522 (11)	
C11	0.9505 (8)	0.4940 (6)	0.62479 (19)	0.0547 (11)	
H11	0.8965	0.5466	0.6554	0.066*	
C12	0.8329 (7)	0.3936 (5)	0.59400 (18)	0.0476 (10)	
H12	0.7010	0.3799	0.6034	0.057*	
C13	1.2739 (10)	0.6246 (7)	0.6464 (2)	0.0714 (15)	
H13A	1.2902	0.7152	0.6248	0.086*	
H13B	1.4014	0.5795	0.6540	0.086*	
H13C	1.2133	0.6468	0.6827	0.086*	
N1	0.7620 (6)	0.0309 (4)	0.55009 (16)	0.0467 (9)	
H1N	0.872 (8)	−0.020 (6)	0.549 (2)	0.056*	
O1	0.8754 (5)	0.1373 (3)	0.46032 (12)	0.0478 (8)	
O2	0.5716 (4)	0.2390 (4)	0.50360 (13)	0.0490 (7)	
Cl1	0.17240 (18)	0.13146 (17)	0.68157 (5)	0.0653 (5)	
Cl2	0.7786 (3)	−0.22328 (19)	0.74746 (6)	0.0826 (6)	
S1	0.76828 (14)	0.18214 (11)	0.51047 (4)	0.0392 (4)	

### Atomic displacement parameters (Å^2^)

**Table:**

	*U*^11^	*U*^22^	*U*^33^	*U*^12^	*U*^13^	*U*^23^
C1	0.049 (2)	0.0334 (18)	0.039 (2)	−0.0094 (16)	0.0126 (16)	−0.0055 (15)
C2	0.051 (2)	0.042 (2)	0.040 (2)	−0.0025 (17)	0.0062 (17)	0.0030 (17)
C3	0.044 (2)	0.045 (2)	0.044 (2)	0.0012 (16)	0.0111 (17)	−0.0025 (18)
C4	0.061 (3)	0.042 (2)	0.038 (2)	−0.0064 (18)	0.0116 (18)	−0.0019 (17)
C5	0.067 (3)	0.039 (2)	0.044 (2)	0.0051 (19)	0.0065 (19)	0.0027 (18)
C6	0.053 (2)	0.041 (2)	0.043 (2)	0.0065 (17)	0.0083 (17)	−0.0036 (17)
C7	0.046 (2)	0.039 (2)	0.0330 (18)	0.0042 (16)	0.0065 (15)	0.0040 (15)
C8	0.048 (2)	0.041 (2)	0.040 (2)	0.0033 (17)	0.0104 (17)	0.0008 (17)
C9	0.052 (3)	0.047 (2)	0.049 (2)	−0.0016 (18)	0.0036 (19)	0.0065 (19)
C10	0.072 (3)	0.040 (2)	0.045 (2)	−0.001 (2)	−0.003 (2)	0.0070 (19)
C11	0.078 (3)	0.050 (2)	0.037 (2)	0.006 (2)	0.008 (2)	−0.0056 (18)
C12	0.055 (3)	0.046 (2)	0.042 (2)	0.0047 (18)	0.0138 (18)	−0.0040 (18)
C13	0.089 (4)	0.068 (3)	0.056 (3)	−0.008 (3)	−0.014 (3)	−0.004 (3)
N1	0.055 (2)	0.0387 (18)	0.047 (2)	0.0038 (15)	0.0219 (17)	0.0029 (15)
O1	0.0598 (18)	0.0507 (17)	0.0337 (14)	0.0098 (13)	0.0148 (12)	−0.0022 (12)
O2	0.0481 (17)	0.0543 (18)	0.0446 (16)	0.0038 (13)	0.0051 (12)	0.0002 (14)
Cl1	0.0525 (7)	0.0842 (9)	0.0604 (7)	0.0132 (6)	0.0205 (5)	0.0067 (6)
Cl2	0.1076 (12)	0.0872 (10)	0.0542 (8)	0.0448 (9)	0.0193 (7)	0.0235 (7)
S1	0.0456 (6)	0.0388 (6)	0.0338 (6)	0.0031 (4)	0.0110 (4)	−0.0010 (4)

### Geometric parameters (Å, °)

**Table:**

C1—C2	1.374 (6)	C8—H8	0.9300
C1—C6	1.384 (6)	C9—C10	1.399 (7)
C1—N1	1.420 (5)	C9—H9	0.9300
C2—C3	1.387 (5)	C10—C11	1.362 (7)
C2—H2	0.9300	C10—C13	1.506 (7)
C3—C4	1.368 (6)	C11—C12	1.379 (7)
C3—Cl1	1.740 (4)	C11—H11	0.9300
C4—C5	1.373 (6)	C12—H12	0.9300
C4—H4	0.9300	C13—H13A	0.9600
C5—C6	1.378 (6)	C13—H13B	0.9600
C5—Cl2	1.746 (5)	C13—H13C	0.9600
C6—H6	0.9300	N1—S1	1.633 (4)
C7—C12	1.380 (6)	N1—H1N	0.87 (5)
C7—C8	1.389 (6)	O1—S1	1.435 (3)
C7—S1	1.753 (4)	O2—S1	1.424 (3)
C8—C9	1.374 (6)		
			
C2—C1—C6	120.5 (4)	C10—C9—H9	119.5
C2—C1—N1	123.1 (4)	C11—C10—C9	118.3 (4)
C6—C1—N1	116.3 (4)	C11—C10—C13	122.0 (5)
C1—C2—C3	118.2 (4)	C9—C10—C13	119.7 (5)
C1—C2—H2	120.9	C10—C11—C12	121.9 (4)
C3—C2—H2	120.9	C10—C11—H11	119.0
C4—C3—C2	123.3 (4)	C12—C11—H11	119.0
C4—C3—Cl1	118.6 (3)	C11—C12—C7	119.3 (4)
C2—C3—Cl1	118.1 (3)	C11—C12—H12	120.4
C3—C4—C5	116.3 (4)	C7—C12—H12	120.4
C3—C4—H4	121.8	C10—C13—H13A	109.5
C5—C4—H4	121.8	C10—C13—H13B	109.5
C4—C5—C6	123.1 (4)	H13A—C13—H13B	109.5
C4—C5—Cl2	118.4 (3)	C10—C13—H13C	109.5
C6—C5—Cl2	118.5 (3)	H13A—C13—H13C	109.5
C5—C6—C1	118.5 (4)	H13B—C13—H13C	109.5
C5—C6—H6	120.7	C1—N1—S1	126.7 (3)
C1—C6—H6	120.7	C1—N1—H1N	113 (4)
C12—C7—C8	120.1 (4)	S1—N1—H1N	112 (4)
C12—C7—S1	120.0 (3)	O2—S1—O1	120.10 (18)
C8—C7—S1	119.8 (3)	O2—S1—N1	108.55 (19)
C9—C8—C7	119.4 (4)	O1—S1—N1	103.68 (17)
C9—C8—H8	120.3	O2—S1—C7	108.50 (19)
C7—C8—H8	120.3	O1—S1—C7	107.86 (19)
C8—C9—C10	120.9 (4)	N1—S1—C7	107.5 (2)
C8—C9—H9	119.5		
			
C6—C1—C2—C3	0.3 (6)	C9—C10—C11—C12	−0.2 (7)
N1—C1—C2—C3	177.7 (4)	C13—C10—C11—C12	−178.1 (5)
C1—C2—C3—C4	−0.6 (7)	C10—C11—C12—C7	0.9 (7)
C1—C2—C3—Cl1	179.2 (3)	C8—C7—C12—C11	−1.4 (6)
C2—C3—C4—C5	0.6 (7)	S1—C7—C12—C11	178.5 (3)
Cl1—C3—C4—C5	−179.1 (3)	C2—C1—N1—S1	37.0 (6)
C3—C4—C5—C6	−0.4 (7)	C6—C1—N1—S1	−145.5 (4)
C3—C4—C5—Cl2	179.6 (3)	C1—N1—S1—O2	−47.9 (4)
C4—C5—C6—C1	0.2 (7)	C1—N1—S1—O1	−176.6 (4)
Cl2—C5—C6—C1	−179.8 (3)	C1—N1—S1—C7	69.3 (4)
C2—C1—C6—C5	−0.2 (6)	C12—C7—S1—O2	37.9 (4)
N1—C1—C6—C5	−177.7 (4)	C8—C7—S1—O2	−142.2 (3)
C12—C7—C8—C9	1.1 (6)	C12—C7—S1—O1	169.5 (3)
S1—C7—C8—C9	−178.7 (3)	C8—C7—S1—O1	−10.7 (4)
C7—C8—C9—C10	−0.4 (6)	C12—C7—S1—N1	−79.3 (4)
C8—C9—C10—C11	0.0 (7)	C8—C7—S1—N1	100.6 (3)
C8—C9—C10—C13	177.9 (4)		

### Hydrogen-bond geometry (Å, °)

**Table:**

*D*—H···*A*	*D*—H	H···*A*	*D*···*A*	*D*—H···*A*
N1—H1N···O1^i^	0.87 (5)	2.02 (6)	2.888 (5)	176 (5)

Symmetry codes: (i) −*x*+2, −*y*, −*z*+1.

## References

[bb1] Enraf–Nonius (1996). *CAD-4-PC* Enraf–Nonius, Delft, The Netherlands.

[bb2] Gelbrich, T., Hursthouse, M. B. & Threlfall, T. L. (2007). *Acta Cryst.* B**63**, 621–632. 10.1107/S010876810701395X 17641433

[bb3] Gowda, B. T., Foro, S., Babitha, K. S. & Fuess, H. (2008). *Acta Cryst.* E**64**, o2190. 10.1107/S1600536808034351 PMC2959669 21581048

[bb4] Gowda, B. T., Foro, S., Nirmala, P. G., Terao, H. & Fuess, H. (2009*a*). *Acta Cryst.* E**65**, o877. 10.1107/S1600536809010459 PMC2969044 21582588

[bb5] Gowda, B. T., Foro, S., Nirmala, P. G., Terao, H. & Fuess, H. (2009*b*). *Acta Cryst.* E**65**, o1219. 10.1107/S1600536809016377 PMC2969678 21583088

[bb6] North, A. C. T., Phillips, D. C. & Mathews, F. S. (1968). *Acta Cryst.* A**24**, 351–359.

[bb7] Perlovich, G. L., Tkachev, V. V., Schaper, K.-J. & Raevsky, O. A. (2006). *Acta Cryst.* E**62**, o780–o782.

[bb8] Sheldrick, G. M. (2008). *Acta Cryst.* A**64**, 112–122. 10.1107/S0108767307043930 18156677

[bb9] Shetty, M. & Gowda, B. T. (2005). *Z. Naturforsch. Teil A*, **60**, 113–120.

[bb10] Spek, A. L. (2009). *Acta Cryst.* D**65**, 148–155. 10.1107/S090744490804362X PMC2631630 19171970

[bb11] Stoe & Cie (1987). *REDU4* Stoe & Cie GmbH, Darmstadt, Germany.

